# OsGLP participates in the regulation of lignin synthesis and deposition in rice against copper and cadmium toxicity

**DOI:** 10.3389/fpls.2022.1078113

**Published:** 2023-01-12

**Authors:** Xiangchao ShangGuan, Ying Qi, Aiguo Wang, Yingnan Ren, Yu Wang, Tengwei Xiao, Zhenguo Shen, Qi Wang, Yan Xia

**Affiliations:** ^1^ College of Life Sciences, Jiangsu Collaborative Innovation Center for Solid Organic Waste Resource, Nanjing Agricultural University, Nanjing, China; ^2^ College of Agronomy, Yunnan Research Center of Urban Agricultural Engineering and Technology, Kunming University, Kunming, China; ^3^ Key Laboratory of Ecological Environment and Tobacco Quality in Tobacco Industry, Zhengzhou Tobacco Research Institute of China National Tobacco Corporation, Zhengzhou, China; ^4^ School of Life Sciences, Zhengzhou University, Zhengzhou, China

**Keywords:** *Oryza sativa* L., germin-like proteins, heavy metal, lignin accumulation, detoxification

## Abstract

Copper (Cu) and cadmium (Cd) are common heavy metal pollutants. When Cd and excessive Cu accumulate in plants, plant growth is reduced. Our previous study showed that Germin-like proteins (GLPs), which exist in tandem on chromosomes, are a class of soluble glycoproteins that respond to Cu stress. In this study, hydroponic cultures were carried out to investigate the effect of GLP on Cd and Cu tolerance and accumulation in rice. The results showed that knockout of a single *OsGLP8*-*2* gene or ten *OsGLP* genes (*OsGLP8*-*2* to *OsGLP8*-*11*) resulted in a similar sensitivity to Cd and Cu toxicity. When subjected to Cu and Cd stress, the *glp8*-*2* and *glp8*-*(2*-*11)* mutants displayed a more sensitive phenotype based on the plant height, root length, and dry biomass of the rice seedlings. Correspondingly, Cu and Cd concentrations in the *glp8*-*2* and *glp8*-*(2*-*11)* mutants were significantly higher than those in the wild-type (WT) and *OsGLP8*-*2*-overexpressing line. However, Cu and Cd accumulation in the cell wall was the opposite. Furthermore, we determined lignin accumulation. The overexpressing-*OsGLP8*-*2* line had a higher lignin accumulation in the shoot and root cell walls than those of the WT, *glp8*-*2*, and *glp8*-*(2*-*11)*. The expression of lignin synthesis genes in the *OsGLP8*-*2*-overexpressing line was significantly higher than that in the WT, *glp8*-*2*, and *glp8*-*(2*-*11)*. The SOD activity of *OsGLP8*-*2*, Diaminobe-nzidine (DAB), propidium iodide (PI) staining, and Malondialdehyde (MDA) content determination suggested that *OsGLP8*-*2* is involved in heavy metal-induced antioxidant defense in rice. Our findings clearly suggest that OsGLPs participate in responses to heavy metal stress by lignin deposition and antioxidant defense capacity in rice, and *OsGLP8-2* may play a major role in the tandem repeat gene clusters of chromosome 8 under heavy metal stress conditions.

## Highlights

- OsGLPs involved in Cd and Cu detoxification and tolerance in rice.
- OsGLPs regulate lignin deposition in cell wall by altering expression of lignin synthesis genes.
- *OsGLP8-2* may play a major role in the tandem repeat gene clusters of rice chromosome 8 when Cd and Cu exposure.

## Introduction

Copper (Cu) is an essential micronutrient element for the normal growth and development of plants (Nazir et al., 2019). However, excessive copper exhibits high toxicity, causing oxidative stress, increasing the reactive oxygen species (ROS) content in plant cells, and destroying the integrity and function of cell membranes (Chrysargyris et al., 2019; Rather et al., 2020). Cadmium (Cd) is a common heavy metal pollutant that is absorbed by plant roots and enters the food chain, endangering human and animal health (Chen et al., 2019a; Zhang et al., 2020). In agriculture, excessive Cu and Cd have many adverse effects on crops, including reducing the germination rate of seeds, changing the growth and morphology of crops, and hindering the absorption of mineral nutrients (Zare et al., 2018; Napoli et al., 2019; Yue et al., 2021; Wang et al., 2021). These adverse effects lead to reduced crop yields and lower quality. The absorption of metal elements by plants is based on plant type and heavy metal. The absorption mechanisms include absorption, transport, accumulation, distribution, rejection, and osmotic adjustment (Ma et al., 2016a).

The cell wall is an important barrier that prevents the transfer of heavy metals into cells (Park and Chon, 2016; Fernández-Fuego et al., 2017; Liu et al., 2019). Previous studies have shown that heavy metals can affect the thickness of plant cell walls, pectin cross-linking, and enzyme activity (Douchiche et al., 2010), thereby affecting cell walls (Zhu et al., 2012; Jia et al., 2021). When subjected to biotic and abiotic stress, lignin metabolism can play a role in stress resistance (Do et al., 2007; Moura et al., 2010; Liu et al., 2018; Wang et al., 2022). Aluminium (Al) can induce lignin synthesis in rice roots, as well as the synthesis of other cell wall components (Mao et al., 2004), and the gene expression of 4-coumarate CoA ligase (4CL), cinnamon alcohol de oxidase (CAD), caffeoyl-CoA-*O*-methyltransferase (CCR), and other enzymes related to lignin synthesis increase (Mao et al., 2004; Mir Derikvand et al., 2008). Cd has a similar effect on soybean growth (Bhuiyan et al., 2007).

Germin-like proteins (GLPs) are a class of soluble glycoproteins that are highly homologous to the germin sequence of wheat (Majeed et al., 2018; Zhang et al., 2018). *GLP* genes have been identified in various plant species (Ilyas et al., 2016). GLPs, most of which are stable oligomers, exist in the extracellular matrix through ionic bonding (Bernier and Berna, 2001; Dunwell et al., 2008). GLPs showed enzymatic activities of oxalate oxidase (OXO), superoxide dismutase (SOD), and polyphenol oxidase (PPO) (Cheng et al., 2014; He et al., 2021). These proteins usually participate in the physiological activities of plants in the form of enzymes, receptors, and structural proteins (Lou and Baldwin, 2006; Dunwell et al., 2008). Earlier studies have shown that *GLPs* are an important class of genes involved in both biotic and abiotic stress responses (Bruno et al., 2014; Zhang et al., 2017; Liao et al., 2021; Zaynab et al., 2022). It has been reported that downregulation of *OsGLP1* sensitised rice to the pathogens of rice blast and sheath blight (Banerjee and Maiti, 2010). Transgenic tobacco plants overexpressing the soybean *GmGLP10* gene displayed enhanced resistance to *Sclerotinia sclerotiorum* infection (Zhang et al., 2017). In addition, these proteins have shown high resistance to salt stress (Hurkman et al., 1991; Barman and Banerjee, 2015; Takeuchi et al., 2016; Banerjee et al., 2017), drought stress (Ke et al., 2009), UV-B radiation (He et al., 2021), and various biological stressors. When exposed to Cu stress, several genes in rice *GLP* family showed higher transcription levels (Li et al., 2016). Similarly, rice treated with Cd also showed higher GLPs abundance (Wei et al., 2021). Zhou et al. (2009) found the GLP protein level of tomato was down-regulated under aluminum stress. However, there are still few studies on the relationship between GLPs and heavy metal tolerance in plants.

A previous study on rice proteomics by immobilised metal ion affinity chromatography-mass spectrometry (IMAC-MS) showed that heavy metal treatment significantly upregulated the abundance of OsGLP proteins (Song et al., 2013) and the transcriptional expression of some members of the *GLP* family (Li et al., 2016). Our current knowledge of the corresponding physiological functions and mechanisms of OsGLPs is still elusive. Here, we hypothesised that *OsGLP* genes are involved in Cd and Cu detoxification in rice. Crispr/Cas-9 technology, which has developed rapidly in recent years, can precisely edit plant genomes and obtain heritable plant material, providing an efficient technical tool for crop genetics (Ma et al., 2016b; Chen et al., 2019b). In this study, *OsGLP* transgenic rice lines, including knockout mutants of the single *OsGLP8*-*2* gene or ten genes (*OsGLP8*-*2 to OsGLP8*-*11*) and overexpressing *OsGLP8*-*2* transgenic rice, were constructed using Crispr/Cas-9 technology and the method of homologous recombination (Court et al., 2002). Furthermore, we functionally characterised OsGLPs responding to Cu and Cd stress in rice through detailed analysis, such as rice phenotype, heavy metal accumulation, lignin deposition in the cell wall, antioxidant defence capacity, and expression of lignin synthesis genes and members of the *OsGLP* family. This study aims to reveal the relationship between rice OsGLPs and plant heavy metal tolerance, and further explains the mechanisms of plant response to heavy metal stress.

## Materials and methods

### Plant materials

Rice seeds were soaked in 10% sodium hypochlorite for 5 min under dark conditions. The seeds were then washed thoroughly, soaked in deionised water, and placed in an incubator at 37°C for germination. Uniformly emerging rice seedlings were evenly placed on a floating net and cultured in 0.5 mol L^−1^ CaCl_2_ nutrient solution in the dark for two days to induce rooting. After CaCl_2_ culture, rice seedlings were cultured with kimura B nutrient solution containing 0.18 mmoL^−1^ KH_2_PO_4_, 0.36 mmoL^−1^ (NH_4_)_2_SO_4_, 0.54 mmoL^−1^ MgSO_4_·7H_2_O, 0.18 mmoL^−1^ KNO_3_, 0.36 mmoL^−1^ Ca(NO_3_)_2_·4H_2_O, 46.25 μmoL^−1^ H_3_BO_3_, 0.32 μmoL^−1^ CuSO_4_·5H_2_O, 0.76 μmoL^−1^ ZnSO_4_·7H_2_O, 9.15 μmoL^−1^ MnCl_2_·4H_2_O, 0.11μmoL^−1^ H_2_MoO_4_·H_2_O, 20 μmoL^−1^ EDTA−FeSO_4_ (pH, 5.6).The nutrient solution was replaced every 2 days.

Two-week-old rice plants were cultured for 5 days under normal conditions, 10 μmol L^−1^ CuSO_4_ treatment, or 25 μmol L^−1^ CdCl_2_ treatment. A normal kimura B nutrient solution was used as the control. Four replicates were set, and each replicate was comprised of 5 rice seedlings. All rice seedlings were grown in a greenhouse under long-day conditions (14 h light/10 h dark) at 28°C/24°C. *Oryza sativa* cv ‘Dongjin’ was used as the wild type (WT) in this study.

### Generation of transgenic plants

The CRISPR/Cas9 system was used to construct the rice mutants. Two sequences of 20 bp in exons of *GLP8*-*2* were selected as gRNAs. Primers were designed based on these sequences, and the annealed product was fused with the pRGEB31 vector. The coding sequences of *OsGLP8*-*2* were amplified from the cDNA of the WT (Dongjin, DJ). Two specific primers (CriOsGLP8-2F and CriOsGLP8-11R) were used to identify mutants, with 10 genes (*OsGLP8*-*2* to *OsGLP8*-*11*) knocked out. If a clear band was observed after 1% agarose gel electrophoresis (**Supplementary Figure S1**), this indicated that the multi-gene knockout mutant was successfully constructed. The coding region sequence of *OsGLP8*-*2* was fused with the pOx vector to form a recombinant plasmid (GLP8-2OE) driven by the 35S promoter. The primers used for vector construction are listed in **Supplementary Table S1**.

The recombinant plasmids were sequenced and introduced into *Agrobacterium tumefaciens* EHA105. The *A*. *tumefaciens*-mediated genetic transformation system was used to construct transgenic rice.

### RNA-seq analysis of *OsGLP* Genes

Four-day-old WT rice seedlings were treated with 3 μmol L^−1^ CuSO_4_ for 12 h. About 50 mg of root tips were collected and snap-frozen in liquid nitrogen to extract total RNA for transcriptome sequencing (GENE DENOVO Biotechnology Co., Ltd, Guangzhou, China). Bioinformatic analysis of the data was performed using the Omicsmart online real-time interactive platform. Each material was repeated three times. Fold change ≥ 2, and FDR ≤ 0.05.

### Determination of Cu and Cd concentrations

The roots of the rice were washed with 20 mmol L^−1^ Na_2_EDTA for 30 min to remove heavy metal ions attached to the surface of the roots. They were then placed in an oven at 80°C until a constant weight, and the dry weight was recorded. Dry plant samples or cell wall materials (0.2 g) were digested with 5.0 mL of guaranteed HNO_3_:HClO_4 =_ 87:13 (*v*:*v*) mixed acid. Cd and Cu concentrations were determined using an inductively coupled plasma optical emission spectrometer (ICP-OES, PerkinElmer, Optima 8000, America). A plant standard [GBW10043 (GSB-21)] was purchased from the National Research Centre for Standards of China and used to ensure reliable results during the digestion and analysis processes.

### Extraction of crude cell walls of rice seedlings

Extraction of crude cell walls was according to the methods of Yang et al. (2011) and Zhu et al. (2020). About 0.5 g of fresh samples of rice shoots and roots were ground with 10 times the volume of 95% ethanol to homogenise them. The mixture was centrifuged at 8,000×*g* for 5 min, and the supernatant was discarded. The pellet was washed 3 times with 95% ethanol. Finally, the pellet was washed twice with ethanol-hexane solution (*v*:*v*=1:2) and dried at room temperature to obtain the crude cell wall. The determination of heavy metals in the cell wall was performed as previously described in Determination of Cu and Cd concentrations section.

### Histochemical and content determination of lignin

The stems of the rice plants were stained with Safranin O-Fast Green staining and paraffin sectioned by embedding technology to determine lignin deposition in the cell walls (Wang et al., 2016; Han et al., 2021). The roots were placed on a Petri dish, and a few drops of 1% phloroglucinol ethanol solution were added. A drop of 35% HCl was added, and the roots were covered with cover glass. After volatilisation and colour development, the roots were placed on a type microscope and magnified four times for observation, and photos were taken.

Six milligrams of cell wall residue were transferred to a glass test tube, and 2.5 mL of 25% bromoacetyl-acetic acid solution (v/v=1:3) and 0.1 mL of 70% perchloric acid were added. The tube was covered, sealed, and placed in a water bath at 70°C for 30 min. The test tube was shaken every 10 min. After cooling, 10 mL of 2 mol L^−1^ NaOH solution was added, and the reaction mixture was diluted to 25 mL with glacial acetic acid. The mixture was centrifuged at 1000×*g* for 5 min. The absorbance of the supernatant was measured at 280 nm, with the reaction solution containing no sample as a blank control.

### qRT-PCR

Total RNA from rice seedlings was extracted using an RNA Extraction Kit (TaKaRa, 9697, China). The cDNA was then obtained after inversion was used as a template, and the SYBR Green fluorescence quantitative kit (TaKaRa, RR420A, China) was used for fluorescence quantitative PCR amplification. The expression of the target gene was calculated using the 2^-ΔΔCt^ method (Gaonkar et al., 2018). The housekeeping gene *ACTIN1* (LOC_Os03g50885) was used as the internal control. Three biological replicates were used for qRT-PCR, and three technical replicates were set for each biological replicate. The primers used are listed in **Supplementary Table S2**.

### Histochemical localisation of H_2_O_2_

The Diaminobenzidine (DAB) staining method was used for the quantitative detection of H_2_O_2_. DAB powder was dissolved in 50 mmol L^−1^ Tris-HCl (pH 6.0) to prepare a 1 mg mL^−1^ dye solution. Rice leaves (3–4 cm) were immersed in DAB dye solution, vacuumed for 2 hours until the leaves sank to the bottom of the tube, and placed in the dark for 12 hours. The dyed rice leaves were boiled in 95% ethanol for decolourisation. After bleaching was complete, the leaves were immersed in 70% ethanol, and pictures were taken using a stereo microscope (Nikon, SMZ1000, Japan).

### Determination of MDA content

Leaf samples (0.1 g) were ground with 1.5 mL of trichloroacetic acid (TCA) on ice and centrifuged at 12,000×*g* for 15 min at 4°C. Then, 500 μL of the supernatant was transferred to a clean 2 mL centrifuge tube, and 1.5 mL of 0.5% thiobarbituric acid (TBA) was added and mixed well. The tubes were placed in a water bath at 90°C for 20 min. After cooling, the mixture was centrifuged at 10,000 rpm for 5 min. The absorbance of the supernatant was determined at 450, 532, and 600 nm. C (μmol L^-1^)=6.45×(A_532_-A_600_)-0.56×A_450_

### Determination of the integrity of the root cell plasma membrane

Rice root tips (1 cm) were placed in 3 μg L^−1^ propidium iodide (PI) solution and soaked in the dark for 15 min. The root tips were removed from the dye solution and rinsed repeatedly with deionised water. Staining was observed with a fluorescence microscope (Zeiss, Axio Imager A1, Germany).

### Statistical analysis

The data were analysed using Excel and SPSS25.0 for analysis of variance and LSD multiple comparison testing (*P* ≤ 0.05). GraphPad Prism 6 was used to graph the data after processing. The values in the graph are the mean ± SD (n = 3). Different letters indicate the differences between several rice lines.

## Results

### Expression of *OsGLP* genes was induced by Cu stress

**Figure 1:**
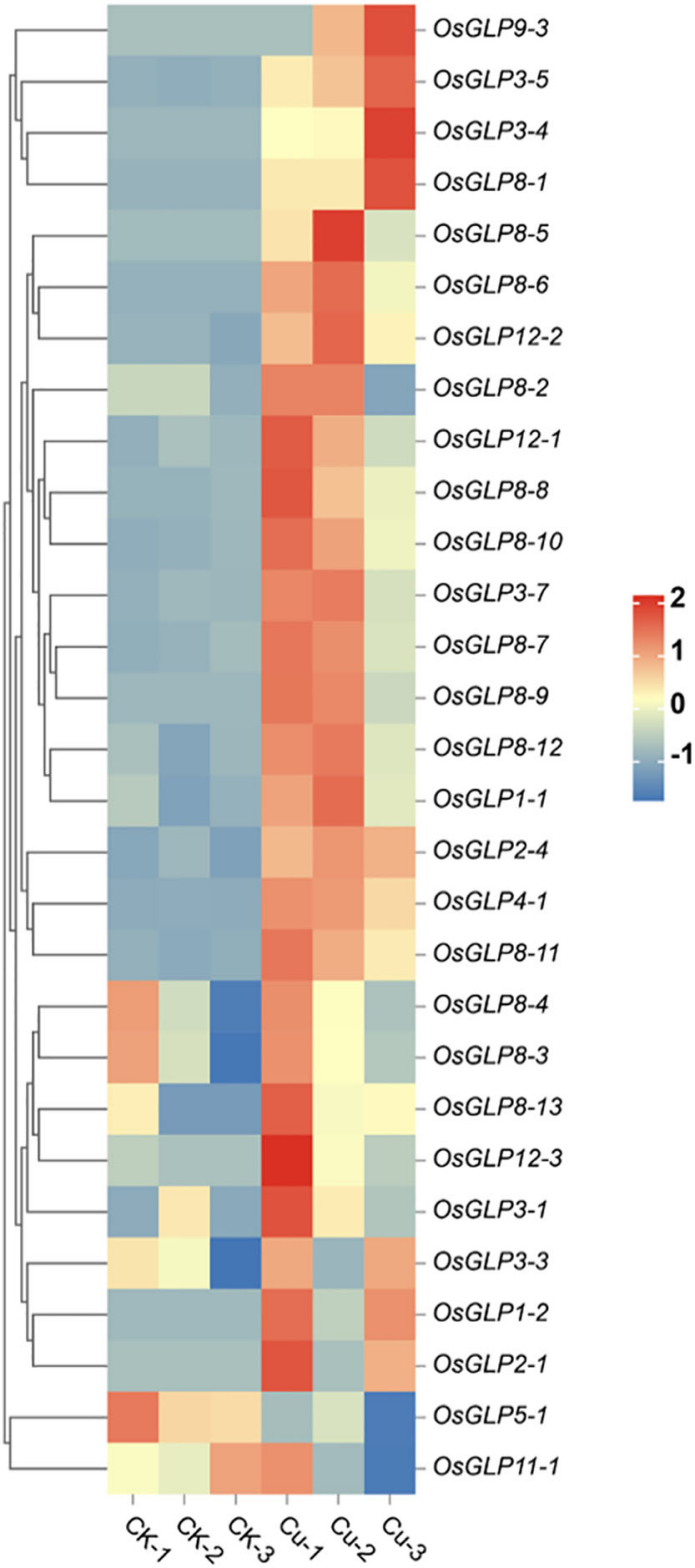
Cu toxicity induces the expression of *OsGLP8*-*2* in rice. Transcription levels of *OsGLPs* increased under heavy metal treatment. Total RNA was isolated from the roots of 4-day-old WT (wild type) rice seedlings treated with 3 μmol L^−1^ CuSO_4_ for 12 h and used for transcriptome sequencing. Values are the mean ± SD; n = 3. Fold change ≥ 2 and FDR ≤ 0.05.

Thirty-two members of the *OsGLP* family were tandem repeat genes and were divided into 8 gene clusters located on chromosomes 1, 2, 3, 8, 9, and 12. Among them, the tandem repeat gene cluster on chromosome 8 was the largest, containing 11 *OsGLP* genes (*OsGLP8*-*1* to *OsGLP8*-*11*) (Li et al., 2016). Four-day-old WT seedlings were treated with 3 μmol L^−1^ CuSO_4_ for 12 h. Total RNA was isolated from rice roots and used for transcriptome sequencing. The heat map showed that the expression of multiple *OsGLP* family genes, such as *OsGLP8*-*2*, *OsGLP8*-*5*, *OsGLP8*-*6*, *OsGLP8*-*7*, *OsGLP8*-*9*, *OsGLP8*-*10*, and *OsGLP8*-*11*, increased significantly after Cu treatment (**Figure 1**). It was inferred that some OsGLPs are Cu-responsive proteins.

### Knockout of single *OsGLP8-2* or 10 *OsGLP* genes exhibits repressed growth

**Figure 2:**
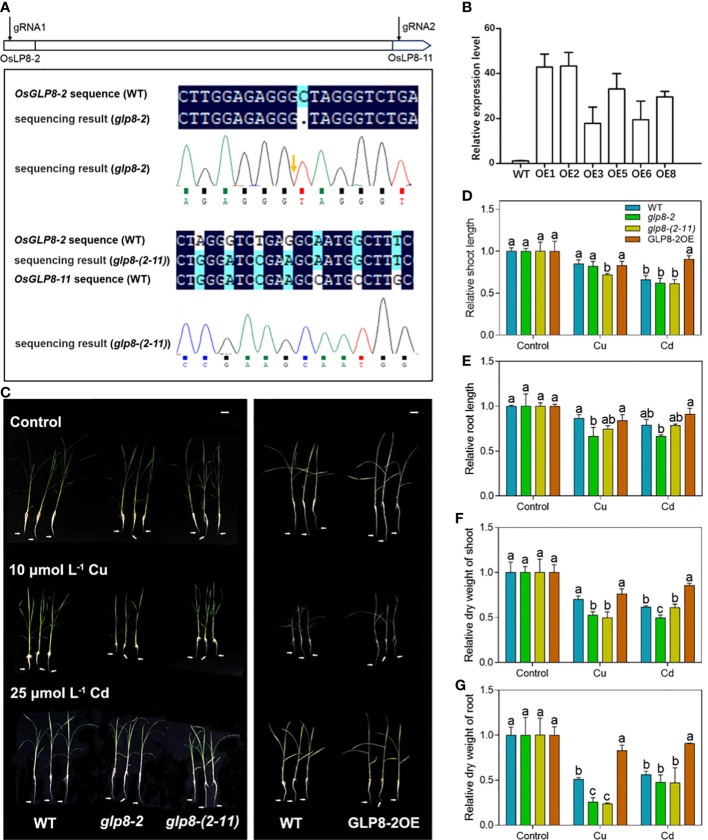
Knockout of O*sGLPs* results in the growth inhibition of rice seedlings. **(A)** Construction and identification of mutants. Two gRNAs were designed based on the exons of *OsGLP8-2* and *OsGLP8-11* near the ATG. Light blue and dark blue indicate successful sequence matching. Wavy lines are sequencing peaks. **(B)** Identification of *OsGLP8*-*2*-overexpressing lines. **(C)** Phenotypes of two-week-old WT (wild type) and transgenic seedlings grown for 5 days under normal conditions, 10 μmol L^−1^ CuSO_4_ treatment, or 25 μmol L^−1^ CdCl_2_ treatment. Scale bar = 3 cm. **(D, E)** Root elongation of WT and transgenic plants. **(F, G)** Dry weight of WT and transgenic plants. Values are the mean ± SD; n = 3. Different letters indicate a difference of *p* ≤ 0.05 by the LSD test.

To understand the contribution of *OsGLPs* to heavy metal tolerance, we designed the primers for *OsGLP8*-*2* and *OsGLP8*-*11* to knock out multiple genes at the same time, and obtained the *glp8*-*2* and *glp8*-*(2*-*11)* mutants, respectively (**Figure 2A**; **Supplementary Figure S2**). Furthermore, we identified 6 overexpression lines and selected GLP8-2OE1 (hereinafter referred to as GLP8-2OE) with the highest expression of *OsGLP8*-*2* for subsequent experiments (**Figure 2B**).

The WT, two mutants *glp8*-*2* and *glp8*-*(2*-*11)*, and GLP8-2OE seedlings were treated with 10 μmol L^−1^ CuSO_4_ or 25 μmol L^−1^ CdCl_2_ for 5 days. Both the *glp8*-*2* and *glp8*-*(2*-*11)* mutants showed hypersensitivity to Cu and Cd toxicity compared with the WT seedlings (**Figure 2C**; **Supplementary Figure S3**). Excess Cu and Cd had a significant inhibitory effect on the shoot height and root elongation of *glp8*-*2* and *glp8*-*(2*-*11)* mutants, while *OsGLP8*-*2* overexpression increased heavy metal tolerance in rice (**Figures 2D, E**). Quantitative analysis further confirmed that the dry weights of *glp8*-*2* and *glp8*-*(2*-*11)* seedlings were significantly lower than those of WT and GLP8-2OE seedlings (**Figures 2F, G**). Overall, *OsGLP* knockout led to a decrease in chlorophyll content (**Supplementary Figure S2**). These results suggest that OsGLPs play an important role in regulating heavy metal tolerance in rice.

### OsGLPs affect Cu and Cd accumulation in rice

**Figure 3:**
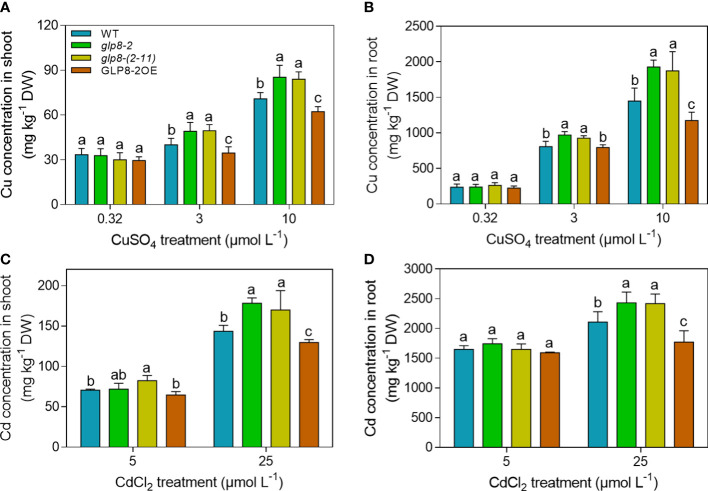
Knockout of OsGLPs affects heavy metal accumulation in rice. **(A, B)** Cu concentrations in the roots and shoots of two-week-old seedlings treated with 3 or 10 μmol L ^−1^ CuSO_4_ for 5 days. **(C, D)** Cd concentrations in the roots and shoots of two-week-old seedlings treated with 5 or 25 μmol L^−1^ CdCl_2_ for 5 days. Values are the mean ± SD; n = 3. Different letters indicate a difference of *p* ≤ 0.05 by the LSD test.

To further investigate the mechanism of OsGLPs regulating heavy metal tolerance in rice, we measured the Cd and Cu concentrations in the shoots and roots, and in those of their cell walls. As shown in **Figure 3**, the Cd and Cu concentrations in both shoots and roots increased significantly with elevated heavy metal levels. At 3 and 10 μmol L^−1^, *glp8*-*2* and *glp8*-*(2*-*11)* seedlings had higher Cu concentrations than the WT, while that in GLP8-2OE was lower. This was especially obvious when the rice seedlings were treated with 10 μmol L^−1^ CuSO_4_ (**Figures 3A, B**). Cd accumulation in different rice seedlings at high levels of Cd (25 μmol L^−1^) displayed a trend similar to Cu toxicity (**Figures 3C, D**). Cd and Cu concentrations in the roots were higher than those in the shoots of the different rice seedlings at the same level of heavy metals.

**Figure 4:**
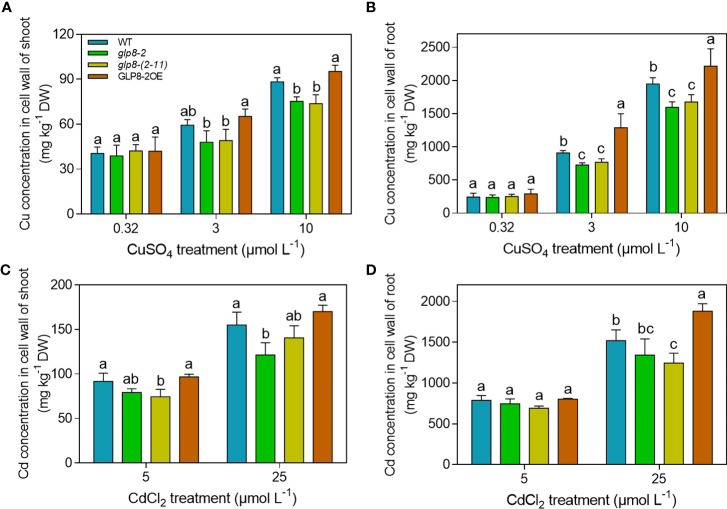
Knockout of OsGLPs affects heavy metal accumulation in the cell walls of rice. **(A, B)** Cu concentrations in the cell walls of roots and shoots of two-week-old seedlings treated with 3 or 10 μmol L ^−1^ CuSO_4_ for 5 days. **(C, D)** Cd concentrations in the cell walls of roots and shoots of two-week-old seedlings treated with 5 or 25 μmol L^−1^ CdCl_2_ for 5 days. Values are the mean ± SD; n = 3. Different letters indicate a difference of *p* ≤ 0.05 by the LSD test.

Cell walls are the main compartments that accumulate heavy metals (Krzeslowska, 2011; Wang et al., 2020; Yan et al., 2022). We determined Cd and Cd concentrations in the cell walls of different rice seedlings to investigate whether OsGLP changes the distribution of heavy metals. In contrast to the results of Cu concentrations in the shoots and roots, the *glp8*-*2* and *glp8*-*(2*-*11)* mutants accumulated less Cu in the cell wall than those of the WT and GLP8-2OE when exposed to 3 and 10 μmol L^−1^ CuSO_4_ (**Figures 4A, B**). Similar to Cu concentration in the cell wall, the loss of *OsGLP8*-*2* resulted in lower Cd retention in the cell wall than the WT and overexpressing rice seedlings at high levels of CdCl_2_ (25 μmol L^−1^) (**Figures 4C, D**). However, there was no obvious difference in heavy metal concentrations of the cell wall between the *glp8*-*2* and *glp8*-(*2*-*11*) rice seedlings.

### OsGLPs affect lignin accumulation in rice

**Figure 5:**
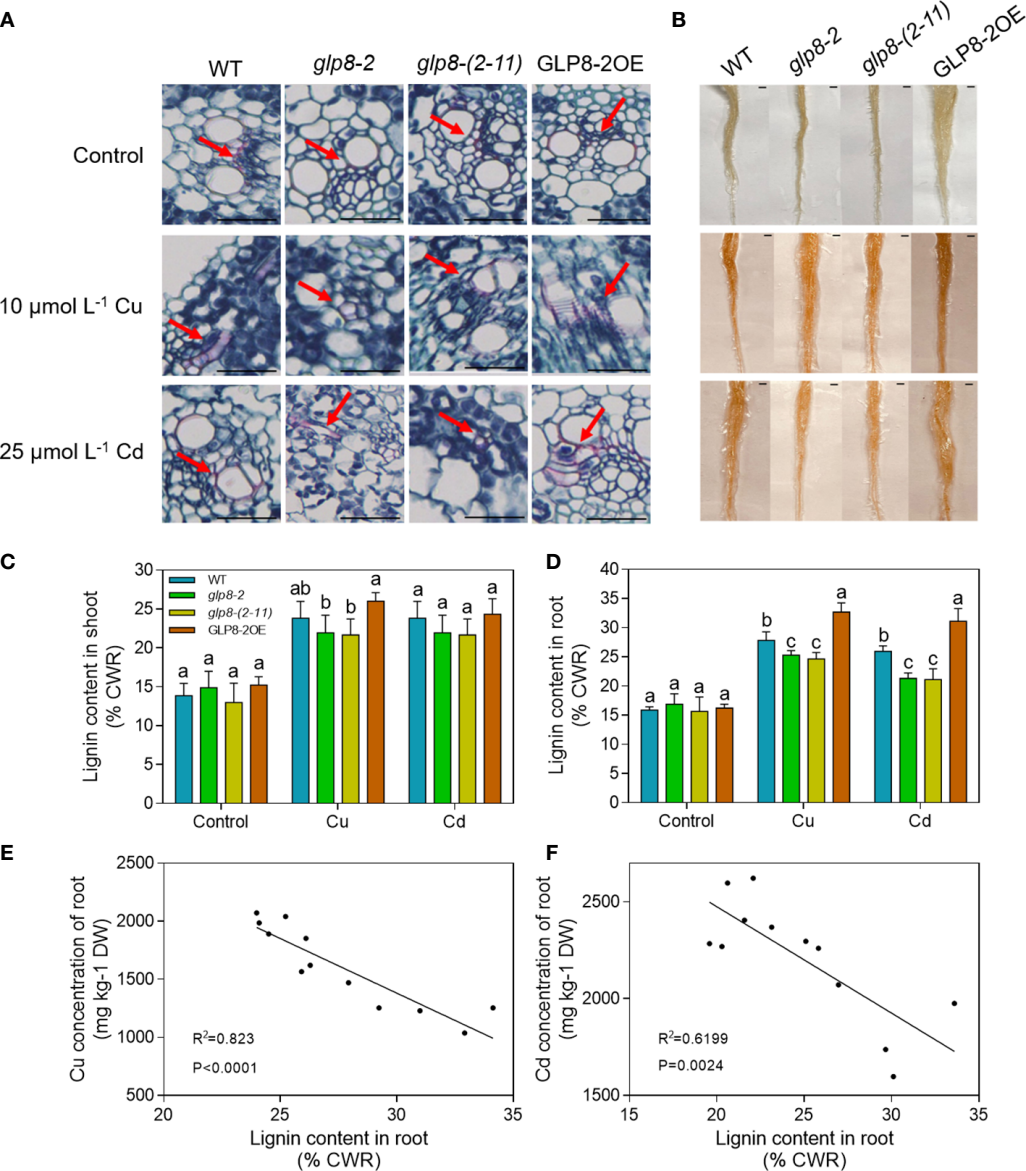
OsGLPs involved in lignin accumulation in rice. **(A)** Effects of Cu or Cd stress on lignin deposition in WT (wild type) and transgenic rice. Seedlings grew for 5 days under normal conditions, 10 μmol L^−1^ CuSO_4_ treatment, or 25 μmol L^−1^ CdCl_2_ treatment. The stems of the rice plants were stained with Safranin O-Fast Green, paraffin embedded, and sectioned. The magnification was 400×. Red indicates that the lignin was successfully dyed. **(B)** Histochemical localisation of lignin in primary roots of two-week-old seedlings grown for 5 days under normal conditions, 10 μmol L^−1^ CuSO_4_ treatment, or 25 μmol L^−1^ CdCl_2_ treatment. The roots were stained with phloroglucinol solution, sliced, and placed on a stereo microscope to take pictures. Scale bar = 1 cm. **(C, D)** The lignin content in the root and shoot cell walls of two-week-old seedlings grown for 5 days under normal conditions, 10 μmol L^−1^ CuSO_4_ treatment, or 25 μmol L^−1^ CdCl_2_ treatment. **(E, F)** Correlation between root Cu/Cd content in rice and lignin content in the root cell walls of rice. These seedlings were treated with 10 μmol L^−1^ CuSO_4_ or 25 μmol L^−1^ CdCl_2_ for 5 days. Values are the mean ± SD; n = 3. Different letters indicate a difference of *p* ≤ 0.05 using the LSD test.

Our previous studies reported that lignin may play a vital role in Cu and Cd stress (Liu et al., 2015; Xia et al., 2018; Su et al., 2020). To investigate the relationships among loss of *OsGLPs*, lignin synthesis, and heavy metal accumulation, we comparatively analysed lignin synthesis in different rice seedlings treated with elevated Cd and Cu levels. The Safranin O-Fast Green staining and phloroglucinol-HCl staining in stems and roots showed that the lignin content in the *glp8*-*2* and *glp8*-*(2*-*11)* mutants was lower than that of the WT and GLP8-2OE rice (**Figures 5A, B**). To further confirm this, we quantitatively determined the lignin content in different rice seedlings treated with Cu and Cd using the acetyl bromide-soluble method (Van Acker et al., 2013; Jang and Lee, 2020). As expected, there was a decrease in the lignin content of the shoots and roots from the two *glp8*-*2* and *glp8*-*(2*-*11)* mutants under Cu and Cd stress, and the lignin content was about 4.6% lower than the WT (**Figures 5C, D**). The lignin content in the roots of OsGLP8-2OE induced by Cu and Cd was higher than that of the WT. A significant negative correlation between the heavy metal concentrations and lignin content was observed in rice roots (*p*< 0.0001 for Cu; *p* = 0.0024 for Cd) (**Figures 5E, F**).

### OsGLPs positively regulate the expression of lignin synthesis-related genes

**Figure 6:**
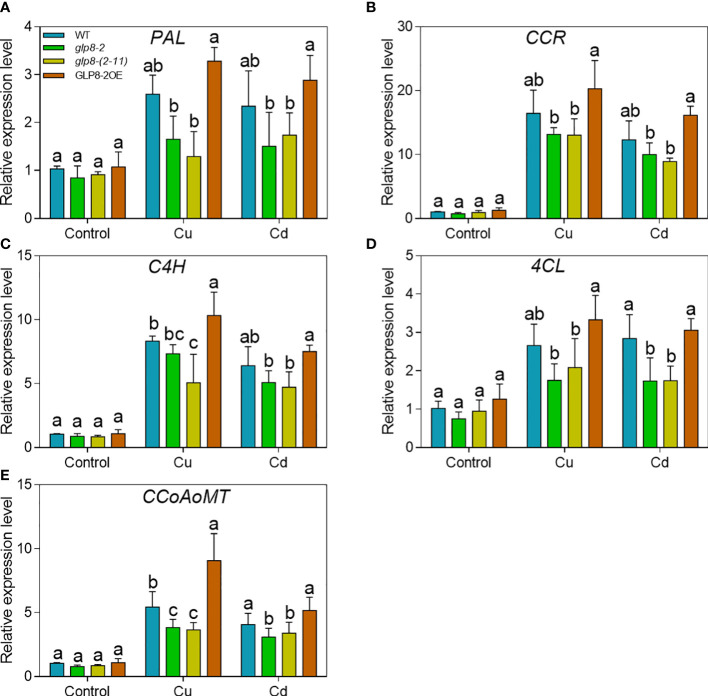
OsGLPs upregulate the expression of lignin synthesis-related genes using RT-qPCR. The expression of *PAL* **(A)**, *CCR* **(B)**, *C4H* **(C)**, *4CL* **(D)**, and *CCoAoMT* **(E)** in 2-week-old WT (wild type) and transgenic seedlings grown for 5 days under normal conditions, 10 μmol L^−1^ CuSO_4_ treatment, or 25 μmol L^−1^ CdCl_2_ treatment. *ACTIN1* (LOC_Os03g50885) was used as the internal control. The relative expression level was obtained by normalisation to the expression level in WT plants without heavy metal treatment. Values are the mean ± SD; n = 3. Different letters indicate a difference of *p* ≤ 0.05 by the LSD test.

To determine whether the alteration of OsGLPs expression levels affects the lignin synthesis pathway, we tested the changes in the expression levels of lignin-related genes, including phenylalanine ammonia-lyase (PAL), 4-coumarate CoA ligase (4CL), caffeoyl-CoA-O-methyltransferase (CCoAoMT), cinnamate-4-hydroxylase (C4H), and cinnamoyl-CoA reductase (CCR). Cu and Cd treatments significantly induced the expression of five lignin biosynthetic enzyme genes (PAL, 4CL, CCoAOMT, C4H, and CCR) (**Figure 6**). When treated with Cu and Cd, the expression levels of these five genes were reduced in the *glp8*-*2* and *glp8*-*(2*-*11)* mutants, especially in the *OsGLP8*-*(2*-*11)* mutants, but elevated significantly in the OsGLP8-2OE seedlings. The expression pattern of five lignin-related genes under Cu and Cd toxicity showed the same trend as lignin content in the roots and heavy metal accumulation in the cell wall.

### OsGLPs participate in heavy metal-induced oxidative damage

**Figure 7:**
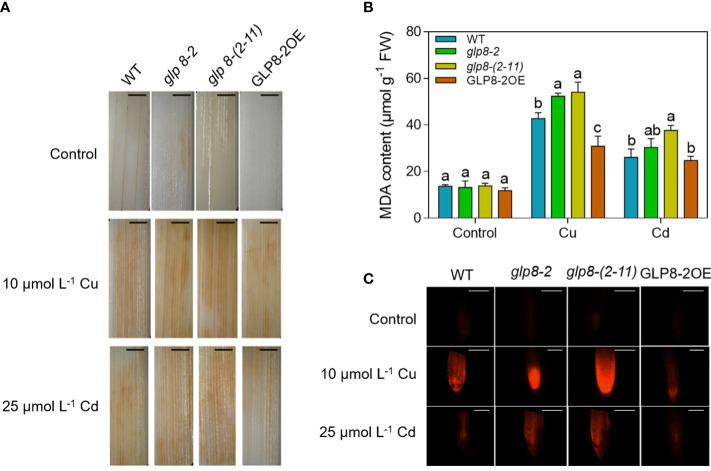
OsGLPs protect against heavy metal-induced oxidative stress. **(A)** Histochemical localisation of H_2_O_2_ in leaves of two-week-old WT (wild type) and transgenic seedlings grown for 5 days under normal conditions, 10 μmol L^−1^ CuSO_4_ treatment, or 25 μmol L^−1^ CdCl_2_ treatment. The shade of brown represents the amount of H_2_O_2_. Scale bar = 2 mm. **(B)** MDA content of leaves of two-week-old WT and transgenic seedlings grown for 5 days under normal conditions, 10 μmol L^−1^ CuSO_4_ treatment, or 25 μmol L^−1^ CdCl_2_ treatment. Values are the mean ± SD; n = 3. Different letters indicate a difference of *p* ≤ 0.05 by the LSD test. **(C)** The integrity of the cell plasma membrane of rice roots of two-week-old WT and transgenic seedlings grown for 5 days under normal conditions, 10 μmol L^−1^ CuSO_4_ treatment, or 25 μmol L^−1^ CdCl_2_ treatment. Red represents the damage to the plasma membrane. The darker the red, the worse the integrity of the plasma membrane. Scale bar = 200 μm.

To explore the role of *OsGLP*s in oxidative stress caused by heavy metal stress, we compared DAB staining in WT and transgenic rice lines. There was no significant difference in the leaf colour of the four lines in the absence of excess Cu and Cd. When exposed to Cu and Cd, the leaf colour was darker than that of the control and GLP8-2OE. Compared with the WT, the *glp8*-*2* and *glp8*-*(2*-*11)* mutants were darker, especially the *OsGLP8*-*(2*-*11)* mutant (**Figure 7A**). This indicates that OsGLPs participate in the elimination of active oxygen in rice cells and can reduce the accumulation of active oxygen caused by heavy metal stress, thereby alleviating the oxidative damage of rice.

Malondialdehyde (MDA) content indicates the degree of peroxidation of the cell membrane and is an important indicator of plant stress resistance (Yang et al., 2019). As shown in **Figure 7B**, Cu and Cd stress aggravated the peroxidation of membrane lipids and increased the MDA content. Loss of the function of OsGLPs led to increased MDA content, which was the opposite of the GLP8-2OE rice seedlings.

Propidium iodide (PI) is a nuclear fluorescent dye that indicates the integrity of the plasma membrane (Li et al., 2021). When the root tip cells are damaged, the permeability of the plasma membrane increases, PI can enter the cell and bind to DNA, and red fluorescence can be observed with a fluorescence microscope. In this study, PI was used to determine the integrity of the plasma membrane in the root tip. Cu and Cd treatments damaged the integrity of the plasma membrane in rice roots. By comparing the intensity of red fluorescence in different rice, the red fluorescence intensity of the *glp8*-*2* and *glp8*-*(2*-*11)* mutants were higher than that of the WT and OsGLP8-2OE (**Figure 7C**) when subjected to Cu and Cd stress, which was consistent with the H_2_O_2_ histochemical localisation and MDA content. This indicates that *OsGLP* genes play a certain role in maintaining the integrity of the plasma membrane.

### OsGLP expression increases under Cu and Cd stress

**Figure 8:**
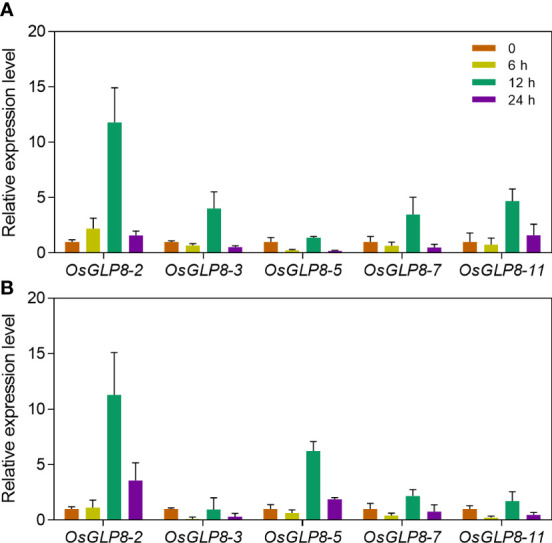
Expression levels of *OsGLPs* induced by heavy metal stress. The expression of *OsGLP8*-*2*, *OsGLP8*-*3*, *OsGLP8*-*5*, *OsGLP8*-*7*, and *OsGLP8*-*11* in two-week-old wild-type seedlings treated with 10 μmol L^−1^ CuSO_4_ **(A)** or 25 μmol L^−1^ CdCl_2_ **(B)** for 0, 6, 12, and 24 hours. Values are the mean ± SD; n = 3. Different letters indicate a difference of *p* ≤ 0.05 by the LSD test.

The knockout mutant of 10 genes (*glp8*-(*2*-*11*)) and the mutant of *OsGLP8*-*2* (*glp8*-*2*) showed the same phenotypes, such as heavy metal tolerance and accumulation, lignin deposition and gene expression levels, and antioxidant defence abilities. We speculated that *OsGLP8*-*2* may display the main contribution in the tandem repeat gene clusters on chromosome 8 in responding to heavy metal stress. The time course for expression levels of *OsGLP8*-*2*, *OsGLP8*-*3*, *OsGLP8*-*5*, *OsGLP8*-*7*, and *OsGLP8*-*11* genes on chromosome 8 were detected under Cu and Cd treatment. The expression of these five genes was significantly upregulated and reached a peak under Cu exposure for 3 h and Cd exposure for 12 h. Among these genes, Cd and Cu treatments upregulated the expression of *OsGLP8*-*2*, with the highest fold change. Its highest level was 71 times higher than that of the control under Cu stress and 11.3 times higher under Cd stress (**Figure 8**). These data demonstrate that OsGLP8-2 is more sensitive to Cu and Cd and is upregulated more than other tandem genes.

## Discussion

Most GLPs have been reported to play three functions in plants, namely as enzymes, structural proteins, and signalling receptors (Bernier and Berna, 2001; Lou and Baldwin, 2006; Pei et al., 2019; Yuan et al., 2021; Zaynab et al., 2022). In this study, transcriptome analysis of rice under Cu stress was performed, in which the transcript levels of multiple genes of the *OsGLP* family showed significant differences compared with the control seedlings (**Figure 1**). Similarly, one member (*OsGLP8-7*) of the *OsGLP* family was identified, and its protein level was significantly upregulated under Cu stress (Chen et al., 2015). We speculated that the increased expression of *OsGLPs* may be a way for plants to cope with heavy metal toxicity.

Based on the unique distribution of the *OsGLP* genes on rice chromosome 8 (Li et al., 2016), gRNAs were designed for *OsGLP8*-*2* and *OsGLP8*-*11* genes to knock out the target genes, and two mutants, *glp8*-*2* and *glp8*-*(2*-*11)*, which were the mutants of the *OsGLP8*-*2* gene and ten genes (*OsGLP8*-*2 to OsGLP8*-*11*), respectively, were obtained. When treated with heavy metals, the loss of function of OsGLPs aggravated growth inhibition in rice (**Figure 2**) and led to higher heavy metal accumulation (**Figure 3**). This suggests that OsGLPs are important in response to heavy metal stress. In fact, studies have reported that two *AtGLP* genes in *Arabidopsis thaliana* L. were induced in large quantities when treated with Cd, indicating that AtGLPs play a role in Cd stress (van de Mortel et al., 2006).

The cell wall is the first barrier for metal ions to enter the plant cytoplasm across the membrane and has a strong ability to fix metal ions (Sun et al., 2013; Gao et al., 2021; Yan et al., 2022). In this study, when subjected to heavy metal stress, the heavy metal concentrations in the cell walls of the *OsGLP* mutants were significantly higher than those of the WT and overexpression line (**Figure 4**). However, the situation was reversed in the whole rice seedlings (**Figure 3**). The loss of OsGLPs led to a higher accumulation of heavy metals in rice; additionally, the ability of the cell wall to retain heavy metals was reduced, and the inward transport of heavy metals was increased. As a result, more heavy metals were enriched in the cytoplasm of the *glp8*-*2* and *glp8*-*(2*-*11)* seedlings, causing more serious toxicity and ultimately leading to a sensitive phenotype. The main components of the cell wall include cellulose, hemicellulose, lignin, and cell wall proteins (Zhao et al., 2019; Roig-Oliver et al., 2020). The abundance of lignin in the cell wall was second only to cellulose. It is a natural macromolecule polymerised by three monolignols: p-hydroxyphenyl (H), guaiacyl (G), and syringyl (S) units. Lignin is essential for maintaining the structural integrity of cell walls and the strength of roots and stems (Slabaugh et al., 2014; Zhao et al., 2020; Zhang et al., 2021). Lignin accumulation increases when plants are exposed to heavy metals, which causes the cell wall to thicken to fix and retard heavy metals, reducing their entry into the cell and causing toxic damage (Moura et al., 2010; Gao et al., 2012; Su et al., 2020; Pan et al., 2021). Therefore, lignin synthesis is a typical defence response of plants to environmental stress. Lignin deposition in the *glp8*-*2* and *glp8*-*(2*-*11)* seedlings was lower than that of the WT and OsGLP8-2OE lines (**Figure 5**). It is generally believed that lignin enhances cell wall rigidification, inhibits root elongation (Xiong et al., 2015). When rice is subjected to heavy metal stress, GLPs can on the one hand increase lignin deposition, thereby inhibiting root growth; on the other hand, it can alleviate heavy metal toxicity, thus promoting root growth. In this study, there was a positive correlation between lignin content and root length, and a negative correlation between lignin content and heavy metal content. When *GLPs* was knocked down, the expression of lignin synthesis-related genes decreased (**Figure 6**). As functional proteins, GLPs may indirectly regulate the expression of these genes by regulating some transcription factors. Therefore, it was inferred that OsGLPs may participate in lignin synthesis.

GLPs mainly have the activities of three enzymes: SOD, OXO, and PPO (Cheng et al., 2014; Ilyas et al., 2020). The function of SOD is to disproportionate O_2_^·-^ into H_2_O_2_ (Smirnoff and Arnaud, 2019). Researchers have shown that OsGLPs are localised to the cell wall. When OsGLPs perform the function of SOD, they cause an increase in the H_2_O_2_ content in the cell wall. The polymerisation of monolignols is the final step in lignin synthesis in the cell wall (Perkins et al., 2022; Zhang et al., 2022). We speculated that OsGLPs could affect lignin biosynthesis through the generated H_2_O_2_. Studies have shown that lignin polymerization is mediated by ROS (Poudel et al., 2019). Removal of H_2_O_2_ with KI (H_2_O_2_ scavenger) resulted in a sharp decrease in extracellular lignin content in *Picea abies* suspension cells (Karkonen et al., 2002). Diaminobenzidine (DAB) is a commonly used chromogenic substrate that reacts with H_2_O_2_ to form a brown precipitate in plant tissue. H_2_O_2_ accumulation can be determined by the shade of the brown substance (Graham and Karnovsky, 1966). However, DAB staining showed that *OsGLP8*-*2* overexpression reduced the H_2_O_2_ content (**Figure 7A**). On the one hand, the measurement of H_2_O_2_ deposition in the cell wall would explain the results better. On the other hand, when excessive H_2_O_2_ disrupts the ROS balance, cells initiate a series of antioxidant enzymes to ensure oxidative homeostasis (Ding et al., 2020). Malondialdehyde (MDA) content indicates the degree of peroxidation of the cell membrane and is an important indicator of plant stress resistance (Yang et al., 2019). Propidium iodide (PI) is a nuclear fluorescent dye that indicates the integrity of the plasma membrane (Li et al., 2021). When the root tip cells are damaged, the permeability of the plasma membrane increases, PI can enter the cell and bind to DNA, and red fluorescence can be observed with a fluorescence microscope. In this study, H_2_O_2_ content, MDA content, and integrity of the plasma membrane in several materials indicate overexpressed GLP8-2 can alleviate the oxidative damage to rice caused by Cu and Cd (**Figure 7**). In addition, *StGLP* overexpression significantly increased the activity of related antioxidant enzymes in potato under heat stress (*Solanum tuberosum* L.) (Gangadhar et al., 2021). These findings imply that overexpression of *GLP* makes antioxidant related physiological activities more active in rice under Cu and Cd stress. Existing studies have shown that oxidation systems in the cell wall, such as peroxidase/H_2_O_2_ and laccase/O_2_^·-^, are not only important components of the antioxidant protection system but also activate the polymerisation of monolignols to generate lignin polymers (Tobimatsu and Schuetz, 2019). When *OsGLP8*-*2* was overexpressed, the cell-wall-localised oxidation system became active, thus enhancing lignin polymerisation. Further studies are needed to confirm the link between heavy metal-induced H_2_O_2_ production and lignin synthesis by H_2_O_2_ scavengers.

In addition, the *glp8*-*2* and *glp8*-*(2*-*11)* mutants did not show significant differences. qRT-PCR of 5 randomly selected genes on chromosome 8 showed that *OsGLP8*-*2* was more easily induced by Cu and Cd, and its upregulation was much greater than other tandem genes (**Figure 8**). This may explain why there was no obvious difference between the *glp8*-*2* and *glp8*-*(2*-*11)* mutants under heavy metal stress conditions.

## Conclusion

Our findings suggest that OsGLPs play a critical role in heavy metal resistance for rice *via* lignin deposition in the cell wall and antioxidant defence capacity. OsGLP8-2 may play a main role in tandem repeat gene clusters on chromosome 8 in rice under heavy metal stress. Further studies on the physiological role of other OsGLP members, except for OsGLP8-2, in this tandem repeat gene cluster should be investigated to explain the diversity of GLP functions.

## Data availability statement

The data presented in the study are deposited in the NCBI SRA repository, accession number PRJNA910469.

## Author contributions

XS: Investigation, writing-original draft. YQ and AW: Investigation and methodology. YW and YR: Investigation. TX: Methodology. ZS: Funding acquisition. QW and YX: Supervision, project administration, writing - review & editing. All authors contributed to the article and approved the submitted version.

## References

[B1] Banerjee J. Gantait S. Maiti M. K. (2017). Physiological role of rice germin-like protein 1 (OsGLP1) at early stages of growth and development in *indica* rice cultivar under salt stress condition. Plant Cell Tiss. Org. 131, 127–137. doi: 10.1073/pnas.1400975111

[B2] Banerjee J. Maiti M. K. (2010). Functional role of rice germin-like protein1 in regulation of plant height and disease resistance. Biochem. Biophy. Res. Commun. 394, 178–183. doi: 10.1016/j.bbrc.2010.02.142 20188068

[B3] Barman A. R. Banerjee J. (2015). Versatility of germin-like proteins in their sequences, expressions, and functions. Funct. Integ R. Genomics 15, 533–548. doi: 10.1007/s10142-015-0454-z 26174051

[B4] Bernier F. Berna A. (2001). Germins and germin-like proteins: Plant do-all proteins. but what do they do exactly? Plant Physiol. Biochem. 39, 545–554. doi: 10.1016/S0981-9428(01)01285-2

[B5] Bhuiyan N. H. Liu W. Liu G. Selvaraj G. Wei Y. King J. (2007). Transcriptional regulation of genes involved in the pathways of biosynthesis and supply of methyl units in response to powdery mildew attack and abiotic stresses in wheat. Plant Mol. Bio. 64, 305–318. doi: 10.1007/s11103-007-9155-x 17406792

[B6] Bruno L. Spadafora N. D. Iaria D. Chiappetta A. Bitonti M. B. (2014). Developmental stimuli and stress factors affect expression of *ClGLP1*, an emerging allergen-related gene in *Citrus limon* . Plant Physio. Biochem. 79, 31–40. doi: 10.1016/j.plaphy.2014.03.003 24681751

[B7] Chen D. Chen D. Xue R. Long J. Lin X. Lin Y.. (2019a). Effects of boron, silicon and their interactions on cadmium accumulation and toxicity in rice plants. J. Hazard. Mater. 367, 447–455. doi: 10.1016/j.jhazmat.2018.12.111 30611037

[B8] Cheng X. Huang X. Liu S. Tang M. Hu W. Pan S. (2014). Characterization of germin-like protein with polyphenol oxidase activity from *Satsuma mandarine* . Biochem. Biophys. Res. Commun. 449, 313–318. doi: 10.1016/j.bbrc.2014.05.027 24845377

[B9] Chen C. Song Y. F. Zhuang K. Li L. Xia Y. Shen Z. G. (2015). Proteomic analysis of copper-binding proteins in excess copper-stressed roots of two rice (*Oryza sativa* l.) varieties with different Cu tolerances. PloS One 10, 0125367. doi: 10.1371/journal.pone.0125367 PMC4412397 25919452

[B10] Chen K. Wang Y. Zhang R. Zhang H. Gao C. (2019b). CRISPR/Cas genome editing and precision plant breeding in agriculture. Annu. Rev. Plant Biol. 70, 667–697. doi: 10.1146/annurev-arplant-050718-100049 30835493

[B11] Chrysargyris A. Papakyriakou E. Petropoulos S. A. Tzortzakis N. (2019). The combined and single effect of salinity and copper stress on growth and quality of *Mentha spicata* plants. J. Hazard. Mater. 368, 584–593. doi: 10.1016/j.jhazmat.2019.01.058 30716568

[B12] Court D. L. Sawitzke J. A. Thomason L. C. (2002). Genetic engineering using homologous recombination. Annu. Rev. Genet. 36, 361–388. doi: 10.1146/annurev.genet.36.061102.093104 12429697

[B13] Ding H. Wang B. Han Y. Li S. (2020). The pivotal function of dehydroascorbate reductase in glutathione homeostasis in plants. J. Exp. Bot. 71, 3405–3416. doi: 10.1093/jxb/eraa107 32107543

[B14] Do C. T. Pollet B. Thévenin J. Sibout R. Denoue D. Barrière Y.. (2007). Both caffeoyl coenzyme a 3-*O*-methyltransferase 1 and caffeic acid *O*-methyltransferase 1 are involved in redundant functions for lignin, flavonoids and sinapoyl malate biosynthesis in arabidopsis. Planta 226, 1117–1129. doi: 10.1007/s00425-007-0558-3 17594112

[B15] Douchiche O. Soret-Morvan O. Chaïbi W. Morvan C. Paynel F. (2010). Characteristics of cadmium tolerance in ‘Hermes’ flax seedlings: Contribution of cell walls. Chemosphere 81, 1430–1436. doi: 10.1016/j.chemosphere.2010.09.011 20884040

[B16] Dunwell J. M. Gibbings J. G. Mahmood T. Naqvi S. M. S. (2008). Germin and germin-like proteins: Evolution, structure, and function. Crit. Rev. Plant Sci. 27, 342–375. doi: 10.1080/07352680802333938

[B17] Fernández-Fuego D. Bertrand A. González A. (2017). Metal accumulation and detoxification mechanisms in mycorrhizal *Betula pubescens* . Environ. pollut. 231, 1153–1162. doi: 10.1016/j.envpol.2017.07.072 28941719

[B18] Gangadhar B. H. Mishra R. K. Kappachery S. Baskar V. Venkatesh J. Nookaraju A.. (2021). Enhanced thermo-tolerance in transgenic potato (*Solanum tuberosum* l.) overexpressing hydrogen peroxide-producing germin-like protein (GLP). Genomics 113, 3224–3234. doi: 10.1016/j.ygeno.2021.07.013 34273496

[B19] Gao M. Y. Chen X. W. Huang W. X. Wu L. Yu Z. S. Xiang L.. (2021). Cell wall modification induced by an arbuscular mycorrhizal fungus enhanced cadmium fixation in rice root. J. Hazard. Mater. 416, 125894. doi: 10.1016/j.jhazmat.2021.125894 34492832

[B20] Gaonkar R. Shiralgi Y. Lakkappa D. B. Hegde G. (2018). Essential oil from *Cymbopogon flexuosus* as the potential inhibitor for HSP90. Toxicol. Rep. 5, 489–496. doi: 10.1016/j.toxrep.2018.03.014 29854620 PMC5978008

[B21] Gao L. Peng K. Chen Y. Wang G. Shen Z. (2012). Roles of apoplastic peroxidases, laccases, and lignification in the manganese tolerance of hyperaccumulator *Phytolacca americana* . Acta Physiol. Plant 34, 151–159. doi: 10.1007/s11738-011-0813-x

[B22] Graham R. C. Karnovsky M. J. (1966). The early stages of absorption of injected horseradish peroxidase in the proximal tubules of mouse kidney: Ultrastructural cytochemistry by a new technique. j. histochem. Cytochem. 14, 291–302. doi: 10.1177/14.4.291 5962951

[B23] Han M. H. Yang N. Wan Q. W. Teng R. Duan A. Q. Wang Y. H.. (2021). Exogenous melatonin positively regulates lignin biosynthesis in *Camellia sinensis* . Int. J. Biol. Macromol. 179, 485–499. doi: 10.1016/j.ijbiomac.2021.03.025 33684430

[B24] He Z. D. Tao M. L. Leung D. W. M. Yan X. Y. Chen L. Peng X. X.. (2021). The rice germin-like protein OsGLP1 participates in acclimation to UV-b radiation. Plant Physiol. 186, 1254–1268. doi: 10.1093/plphys/kiab125 33713137 PMC8195522

[B25] Hurkman W. J. Tao H. P. Tanaka C. K. (1991). Germin-like polypeptides increase in barley roots during salt stress. Plant Physiol. 97, 366–374. doi: 10.1104/pp.97.1.366 16668394 PMC1081007

[B26] Ilyas M. Irfan M. Mahmood T. Hussain H. Latif ur R. Naeem I.. (2020). Analysis of germin-like protein genes (OsGLPs) family in rice using various in silico approaches. Curr. Bioinform. 15, 17–33. doi: 10.2174/1574893614666190722165130

[B27] Ilyas M. Rasheed A. Mahmood T. (2016). Functional characterization of germin and germin-like protein genes in various plant species using transgenic approaches. Biotechnol. Lett. 38, 1405–1421. doi: 10.1007/s10529-016-2129-9 27230937

[B28] Jang J. H. Lee O. R. (2020). Overexpression of ginseng patatin-related phospholipase *pPLAIIIβ* alters the polarity of cell growth and decreases lignin content in *Arabidopsis* . J. Ginseng Res. 44, 321–331. doi: 10.1016/j.jgr.2019.01.004 32148415 PMC7031755

[B29] Jia H. L. Wang X. H. Wei T. Wang M. Liu X. Hua L.. (2021). Exogenous salicylic acid regulates cell wall polysaccharides synthesis and pectin methylation to reduce cd accumulation of tomato. Ecotox. Environ. Safe. 207, 111550. doi: 10.1016/j.ecoenv.2020.111550 33254408

[B30] Karkonen A. Koutaniemi S. Mustonen M. Syrjanen K. Brunow G. Kilpelainen I.. (2002). Lignification related enzymes in *Picea abies* suspension cultures. Physiol. Plantarum 114, 343–353. doi: 10.1034/j.1399-3054.2002.1140303.x 12060256

[B31] Ke Y. Han G. He H. Li J. (2009). Differential regulation of proteins and phosphoproteins in rice under drought stress. Biochem. Biophys. Res. Commun. 379, 133–138. doi: 10.1016/j.bbrc.2008.12.067 19103168

[B32] Krzeslowska M. (2011). The cell wall in plant cell response to trace metals: Polysaccharide remodeling and its role in defense strategy. Acta Physiol. Plant 33, 35–51. doi: 10.1007/s11738-010-0581-z

[B33] Liao L. T. Hu Z. Y. Liu S. Q. Yang Y. G. Zhou Y. (2021). Characterization of germin-like proteins (GLPs) and their expression in response to abiotic and biotic stresses in cucumber. Hortic. 7, 412. doi: 10.3390/horticulturae7100412

[B34] Liu Q. Luo L. Zheng L. (2018). Lignins: biosynthesis and biological functions in plants. Int. J. Mol. Sci. 19, 335. doi: 10.3390/ijms19020335 29364145 PMC5855557

[B35] Liu Y. Lv H. Yang N. Li Y. Liu B. Rensing C.. (2019). Roles of root cell wall components and root plaques in regulating elemental uptake in rice subjected to selenite and different speciation of antimony. Environ. Exp. Bot. 163, 36–44. doi: 10.1016/j.envexpbot.2019.04.005

[B36] Liu Q. Q. Zheng L. He F. Zhao F. J. Shen Z. G. Zheng L. Q. (2015). Transcriptional and physiological analyses identify a regulatory role for hydrogen peroxide in the lignin biosynthesis of copper-stressed rice roots. Plant Soil 387, 323–336. doi: 10.1007/s11104-014-2290-7

[B37] Li L. Xu X. Chen C. Shen Z. (2016). Genome-wide characterization and expression analysis of the germin-like protein family in rice and arabidopsis. Int. J. Mol. Sci. 17, 1622. doi: 10.3390/ijms17101622 27669230 PMC5085655

[B38] Li Q. Zhao Y. Zhu X. M. Xie Y. L. (2021). Antifungal efficacy of paeonol on *Aspergillus flavus* and its mode of action on cell walls and cell membranes. LWT-Food Sci. Technol. 149, 111985. doi: 10.1016/j.lwt.2021.111985

[B39] Lou Y. Baldwin I. T. (2006). Silencing of a germin-like gene in *Nicotiana attenuata* improves performance of native herbivores. Plant Physiol. 140, 1126–1136. doi: 10.1104/PP.105.073700 16461381 PMC1400569

[B40] Majeed N. Javaid B. Deeba F. Naqvi S. M. S. Douches D. S. (2018). Enhanced *fusarium oxysporum* f. sp. *tuberosi* resistance in transgenic potato expressing a rice GLP superoxide dismutase gene. Am. J. Potato Res. 95, 383–394. doi: 10.1007/s12230-018-9639-z

[B41] Mao C. Yi K. Yang L. Zheng B. Wu Y. Liu F.. (2004). Identification of aluminium-regulated genes by cDNA-AFLP in rice (*Oryza sativa* l.): Aluminium-regulated genes for the metabolism of cell wall components. J. Exp. Bot. 55, 137–143. doi: 10.1093/jxb/erh030 14645395

[B42] Ma Y. Rajkumar M. Zhang C. Freitas H. (2016a). Inoculation of *Brassica oxyrrhina* with plant growth promoting bacteria for the improvement of heavy metal phytoremediation under drought conditions. J. Hazard. Mater. 320, 36–44. doi: 10.1016/j.jhazmat.2016.08.009 27508309

[B43] Ma X. Zhu Q. Chen Y. Liu Y. G. (2016b). CRISPR/Cas9 platforms for genome editing in plants: Developments and applications. Mol. Plant 9, 961–974. doi: 10.1016/j.molp.2016.04.009 27108381

[B44] Mir Derikvand M. Sierra J. B. Ruel K. Pollet B. Do C. T. Thévenin J.. (2008). Redirection of the phenylpropanoid pathway to feruloyl malate in *Arabidopsis* mutants deficient for cinnamoyl-CoA reductase 1. Planta 227, 943–956. doi: 10.1007/s00425-007-0669-x 18046574

[B45] Moura J. C. M. S. Bonine C. A. V. de Oliveira Fernandes Viana J. Dornelas M. C. Mazzafera P. (2010). Abiotic and biotic stresses and changes in the lignin content and composition in plants. J. Integr. Plant Biol. 52, 360–376. doi: 10.1111/j.1744-7909.2010.00892.x 20377698

[B46] Napoli M. Cecchi S. Grassi C. Baldi A. Zanchi C. A. Orlandini S. (2019). Phytoextraction of copper from a contaminated soil using arable and vegetable crops. Chemosphere 219, 122–129. doi: 10.1016/j.chemosphere.2018.12.017 30537585

[B47] Nazir F. Hussain A. Fariduddin Q. (2019). Hydrogen peroxide modulate photosynthesis and antioxidant systems in tomato (*Solanum lycopersicum* l.) plants under copper stress. Chemosphere 230, 544–558. doi: 10.1016/j.chemosphere.2019.05.001 31125883

[B48] Pan J. Guan M. Xu P. Chen M. Cao Z. (2021). Salicylic acid reduces cadmium (Cd) accumulation in rice (*Oryza sativa* l.) by regulating root cell wall composition *via* nitric oxide signaling. Sci. Total Environ. 797, 149202. doi: 10.1016/j.scitotenv.2021.149202 34346363

[B49] Park J. H. Chon H. T. (2016). Characterization of cadmium biosorption by *Exiguobacterium* sp isolated from farmland soil near Cu-Pb-Zn mine. Environ. Sci. pollut. Res. 23, 11814–11822. doi: 10.1007/s11356-016-6335-8 26951224

[B50] Pei Y. K. Li X. C. Zhu Y. T. Ge X. Y. Sun Y. Liu N. N.. (2019). GhABP19, a novel germin-like protein from *Gossypium hirsutum*, plays an important role in the regulation of resistance to verticillium and fusarium wilt pathogens. Front. Plant Sci. 10. doi: 10.3389/fpls.2019.00583 PMC6517559 31134119

[B51] Perkins M. L. Schuetz M. Unda F. Chen K. T. Bally M. B. Kulkarni J. A.. (2022). Monolignol export by diffusion down a polymerization-induced concentration gradient. Plant Cell 34, 2080–2095. doi: 10.1093/plcell/koac051 35167693 PMC9048961

[B52] Poudel A. Navathe S. Chand R. Mishra V. K. Singh P. K. Joshi A. K. (2019). Hydrogen peroxide prompted lignification affects pathogenicity of hemi-biotrophic pathogen *Bipolaris sorokiniana* to wheat. Plant Pathol. J. 35, 287–300. doi: 10.5423/PPJ.OA.09.2018.0180 31481852 PMC6706009

[B53] Rather B. A. Masood A. Sehar Z. Majid A. Anjum N. A. Khan N. A. (2020). Mechanisms and role of nitric oxide in phytotoxicity-mitigation of copper. Front. Plant Sci. 11. doi: 10.3389/fpls.2020.00675 PMC7274197 32547583

[B54] Roig-Oliver M. Bresta P. Nadal M. Liakopoulos G. Nikolopoulos D. Karabourniotis G.. (2020). Cell wall composition and thickness affect mesophyll conductance to CO_2_ diffusion in *Helianthus annuus* under water deprivation. J. Exp. Bot. 71, 7198–7209. doi: 10.1093/jxb/eraa413 32905592

[B55] Slabaugh E. Davis J. K. Haigler C. H. Yingling Y. G. Zimmer J. (2014). Cellulose synthases: New insights from crystallography and modeling. Trends. Plant Sci. 19, 99–106. doi: 10.1016/j.tplants.2013.09.009 24139443

[B56] Smirnoff N. Arnaud D. (2019). Hydrogen peroxide metabolism and functions in plants. N. Phytol. 221, 1197–1214. doi: 10.1111/nph.15488 30222198

[B57] Song Y. F. Cui J. Zhang H. X. Wang G. P. Zhao F. J. Shen Z. G. (2013). Proteomic analysis of copper stress responses in the roots of two rice (*Oryza sativa l.*) varieties differing in Cu tolerance. Plant Soil 366, 647–658. doi: 10.1007/s11104-012-1458-2

[B58] Su N. N. Ling F. Xing A. M. Zhao H. H. Zhu Y. W. Wang Y.. (2020). Lignin synthesis mediated by CCoAOMT enzymes is required for the tolerance against excess Cu in *Oryza sativa* . Environ. Exp Bot. 175, 104059. doi: 10.1016/j.envexpbot.2020.104059

[B59] Sun J. Cui J. Luo C. Gao L. Chen Y. Shen Z. (2013). Contribution of cell walls, nonprotein thiols, and organic acids to cadmium resistance in two cabbage varieties. Arch. Environ. Contam. Toxicol. 64, 243–252. doi: 10.1007/s00244-012-9824-x 23111495

[B60] Takeuchi K. Hasegawa H. Gyohda A. Komatsu S. Okamoto T. Okada K.. (2016). Overexpression of *RSOsPR10*, a root-specific rice PR10 gene, confers tolerance against drought stress in rice and drought and salt stresses in bentgrass. Plant Cell Tiss. Org. 127, 35–46. doi: 10.1007/s11240-016-1027-0

[B61] Tobimatsu Y. Schuetz M. (2019). Lignin polymerization: How do plants manage the chemistry so well? Curr. Opin. Biotechnol. 56, 75–81. doi: 10.1016/j.copbio.2018.10.001 30359808

[B62] Van Acker R. Vanholme R. Storme V. Mortimer J. C. Dupree P. Boerjan W. (2013). Lignin biosynthesis perturbations affect secondary cell wall composition and saccharification yield in *Arabidopsis thaliana* . Biotechnol. Biofuels 6, 46. doi: 10.1186/1754-6834-6-46 23622268 PMC3661393

[B63] van de Mortel J. E. Almar Villanueva L. Schat H. Kwekkeboom J. Coughlan S. Moerland P. D.. (2006). Large Expression differences in genes for iron and zinc homeostasis, stress response, and lignin biosynthesis distinguish roots of *Arabidopsis thaliana* and the related metal hyperaccumulator *Thlaspi caerulescens* . Plant Physiol. 142, 1127–1147. doi: 10.1104/pp.106.082073 16998091 PMC1630723

[B64] Wang X. S. Fu H. I. Gong F. Y. Zhang Y. He C. Y. Yang Z. Y. (2020). Lignin side chain region participates in cd detoxification related to the cultivar-dependent cd accumulation in *Brassica chinensis* l. J. Hazard. Mater. 392, 122264. doi: 10.1016/j.jhazmat.2020.122264 32078971

[B65] Wang Y. Gui C. Wu J. Gao X. Huang T. Cui F.. (2022). Spatio-temporal modification of lignin biosynthesis in plants: A promising strategy for lignocellulose improvement and lignin valorization. Fron. Bioeng. Biotechnol. 10. doi: 10.3389/fbioe.2022.917459 PMC9283729 35845403

[B66] Wang G. L. Huang Y. Zhang X. Y. Xu Z. S. Wang F. Xiong A. S. (2016). Transcriptome-based identification of genes revealed differential expression profiles and lignin accumulation during root development in cultivated and wild carrots. Plant Cell Rep. 35, 1743–1755. doi: 10.1007/s00299-016-1992-0 27160835

[B67] Wang M. Hu C. Xu J. Jing X. Rahim H. U. Cai X. (2021). Facile combinations of thiosulfate and zerovalent iron synergically immobilize cadmium in soils through mild extraction and facilitated immobilization. J. Hazard. Mater. 407, 124806. doi: 10.1016/j.jhazmat.2020.124806 33341570

[B68] Wei W. Peng H. Xie Y. Wang X. Huang R. Chen H.. (2021). The role of silicon in cadmium alleviation by rice root cell wall retention and vacuole compartmentalization under different durations of cd exposure. Ecotox. Environ. Safe. 226, 112810. doi: 10.1016/j.ecoenv.2021.112810 34571424

[B69] Xia Y. Liu J. Wang Y. Zhang X. X. Shen Z. G. Hu Z. B. (2018). Ectopic expression of *Vicia sativa* caffeoyl-CoA *O*-methyltransferase (*VsCCoAOMT*) increases the uptake and tolerance of cadmium in arabidopsis. Environ. Exp. Bot. 145, 47–53. doi: 10.1016/j.envexpbot.2017.10.019

[B70] Xiong J. Yang Y. Fu G. Tao L. (2015). Novel roles of hydrogen peroxide (H_2_O_2_) in regulating pectin synthesis and demethylesterification in the cell wall of rice (*Oryza sativa*) root tips. New Phytol. 206, 118–126. doi: 10.1111/nph.13285 25615266

[B71] Yang J. Lin L. N. Zhang X. B. Wu Y. Bi B. Zhang G. C.. (2019). Sodium pheophorbide a has photoactivated fungicidal activity against *Pestalotiopsis neglecta* . Pest. Biochem. Physiol. 158, 25–31. doi: 10.1016/j.pestbp.2019.04.003 31378357

[B72] Yang J. L. Zhu X. F. Peng Y. X. Zheng C. Li G. X. Liu Y.. (2011). Cell wall hemicellulose contributes significantly to aluminum adsorption and root growth in. Arabidopsis. Plant Physiol. 155, 1885–1892. doi: 10.1104/pp.111.172221 21285327 PMC3091086

[B73] Yan L. Li S. Cheng J. Zhang Y. Jiang C. (2022). Boron-mediated lignin metabolism in response to aluminum toxicity in citrus (*Poncirus trifoliata* (L.) raf.) root. Plant Physiol. Bioch. 185, 1–12. doi: 10.1016/j.plaphy.2022.05.018 35640496

[B74] Yuan B. Yang Y. Fan P. Liu J. Xing H. Liu Y.. (2021). Genome-wide identification and characterization of germin and germin-like proteins (GLPs) and their response under powdery mildew stress in wheat (*Triticum aestivum* l.). Plant Mol. Biol. Rep. 39, 821–832. doi: 10.1007/s11105-021-01291-w

[B75] Yue Z. Chen Y. Chen C. Ma K. Tian E. Wang Y.. (2021). Endophytic *Bacillus altitudinis* WR10 alleviates Cu toxicity in wheat by augmenting reactive oxygen species scavenging and phenylpropanoid biosynthesis. J. Hazard. Mater. 405, 124272. doi: 10.1016/j.jhazmat.2020.124272 33097348

[B76] Zare A. A. Khoshgoftarmanesh A. H. Malakouti M. J. Bahrami H. A. Chaney R. L. (2018). Root uptake and shoot accumulation of cadmium by lettuce at various Cd:Zn ratios in nutrient solution. Ecotox. Environ. Safe. 148, 441–446. doi: 10.1016/j.ecoenv.2017.10.045 29102904

[B77] Zaynab M. Peng J. Sharif Y. Fatima M. Albaqami M. Al-Yahyai R.. (2022). Genome-wide identification and expression profiling of germin-like proteins reveal their role in regulating abiotic stress response in potato. Front. Plant Sci. 12. doi: 10.3389/fpls.2021.831140 PMC8891383 35251067

[B78] Zhang Q. Cai W. Ji T. T. Ye L. Lu Y. T. Yuan T. T. (2020). WRKY13 enhances cadmium tolerance by promoting *D-CYSTEINE DESULFHYDRASE* and hydrogen sulfide production1. Plant Physiol. 183, 345–357. doi: 10.1104/pp.19.01504 32179630 PMC7210638

[B79] Zhang N. Guan R. Yang Y. Bai Z. Ge F. Liu D. (2017). Isolation and characterization of a *Fusarium oxysporum*-resistant gene *LrGLP1* from *Lilium regale* Wilson. In Vitro Cell. Dev. Biol. Plant 53, 461–468. doi: 10.1007/s11627-017-9829-2

[B80] Zhang J. Liu Y. Li C. Yin B. Liu X. Guo X.. (2022). PtomtAPX is an autonomous lignification peroxidase during the earliest stage of secondary wall formation in *Populus tomentosa* Carr. Nat. Plants 8, 828–839. doi: 10.1038/s41477-022-01181-3 35851622

[B81] Zhang Y. Wang X. Chang X. Sun M. Zhang Y. Li W.. (2018). Overexpression of germin-like protein GmGLP10 enhances resistance to *Sclerotinia sclerotiorum* in transgenic tobacco. Biochem. Biophys. Res. Commun. 497, 160–166. doi: 10.1016/j.bbrc.2018.02.046 29428735

[B82] Zhang Q. Wang L. Wang Z. Zhang R. Liu P. Liu M.. (2021). The regulation of cell wall lignification and lignin biosynthesis during pigmentation of winter jujube. Hortic. Res. 8, 238. doi: 10.1038/s41438-021-00670-4 34719675 PMC8558337

[B83] Zhao D. Luan Y. Xia X. Shi W. Tang Y. Tao J. (2020). Lignin provides mechanical support to herbaceous peony (*Paeonia lactiflora* pall.) stems. Hortic. Res. 7, 213. doi: 10.1038/s41438-020-00451-5 33372177 PMC7769982

[B84] Zhao Y. Y. Man Y. Wen J. L. Guo Y. Y. Lin J. X. (2019). Advances in imaging plant cell walls. Trends Plant Sci. 24, 867–878. doi: 10.1016/j.tplants.2019.05.009 31257154

[B85] Zhou S. Sauvé R. Thannhauser T. W. (2009). Proteome changes induced by aluminium stress in tomato roots. J. Exp. Bot. 60, 1849–1857. doi: 10.1093/jxb/erp065 19336389

[B86] Zhu X. F. Lei G. J. Jiang T. Liu Y. Li G. X. Zheng S. J. (2012). Cell wall polysaccharides are involved in p-deficiency-induced cd exclusion in *Arabidopsis thaliana* . Planta 236, 989–997. doi: 10.1007/s00425-012-1652-8 22526505

[B87] Zhu X. F. Wu Q. Meng Y. T. Tao Y. Shen R. F. (2020). AtHAP5A regulates iron translocation in iron-deficient *Arabidopsis thaliana* . J. Integr. Plant Biol. 62, 1910–1925. doi: 10.1111/jipb.12984 33405355

