# Supplementary material for: OsGLP participates in the regulation of lignin synthesis and deposition in rice against copper and cadmium toxicity
Source: Front Plant Sci. 2023 Jan 12;13:1078113. doi: 10.3389/fpls.2022.1078113 (PMC9878301; doi:10.3389/fpls.2022.1078113)
Supplement: Supplementary file 1 [file DataSheet_1.pdf]

Supplementary Materials

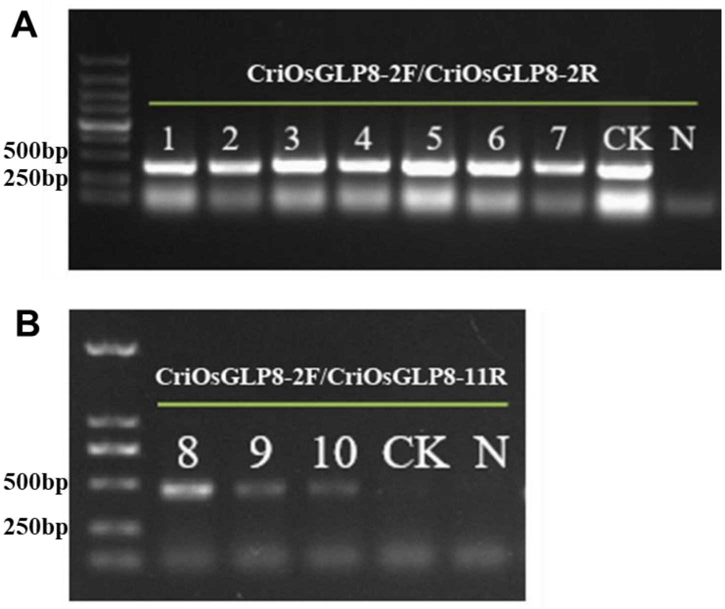

FIGURE S1 | Construction of transgenic rice lines. **(A)** Validation of *osglp8-2* mutant and **(B)** Validation of *osglp8-(2-11)* mutant. CK means control check, N means negative control.

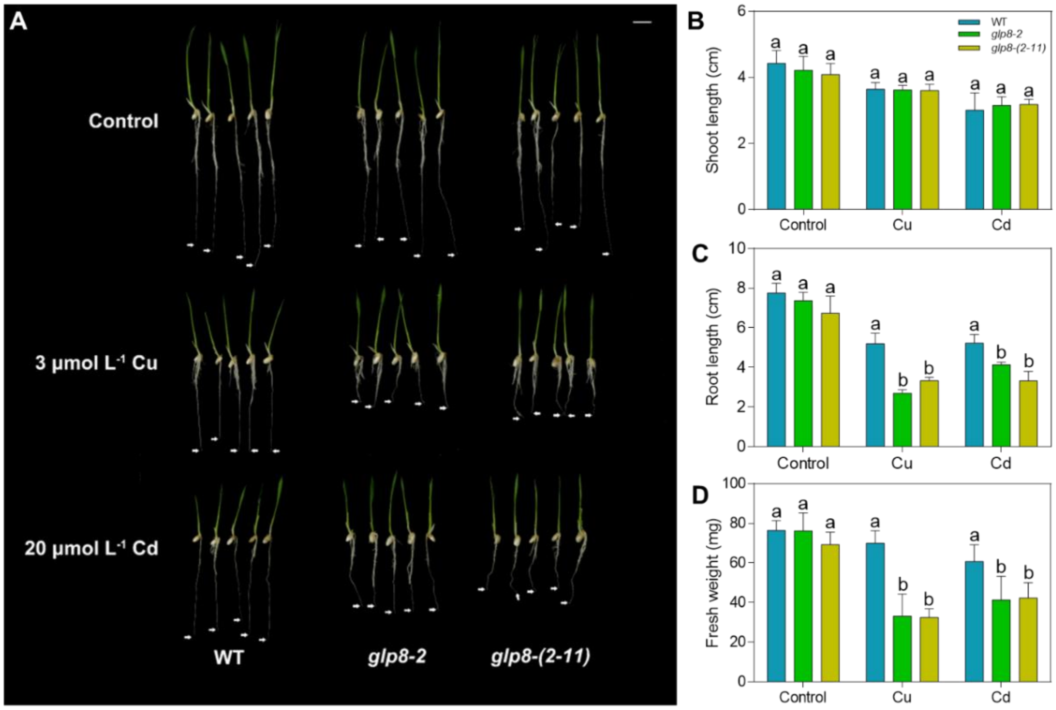

FIGURE S2 | Knockout of O*sGLPs* results in the growth inhibition of rice seedlings. **(A)** Phenotypes of 4-day-old WT and transgenic seedlings grown for 3 days under normal conditions, 3 μmol L^{-1} $CuSO_4$ treatment, or 20 μmol L^{-1} $CdCl_2$ treatment. Scale bar = 1 cm. **(B and C)** Root elongation of WT (wild type) and transgenic plants. (**D**) Fresh weight of WT and transgenic plants. Values are the mean ± SD; n = 3. Different letters indicate a difference of $p \leq 0.05$ by the LSD test.

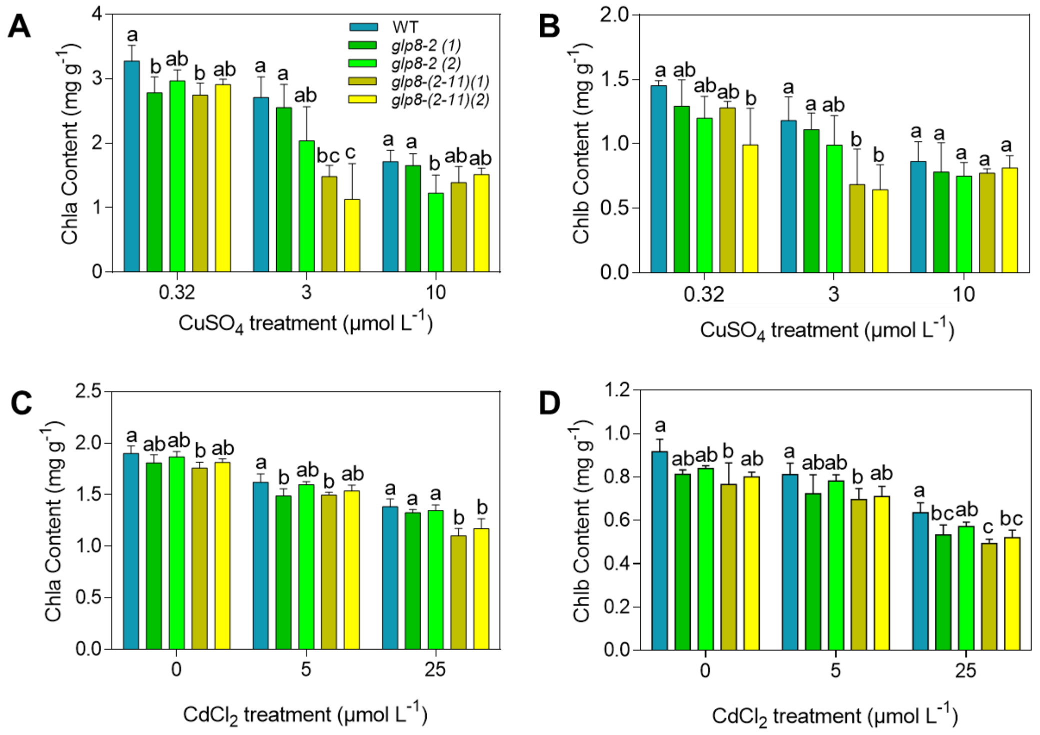

FIGURE S3 | Knockout of OsGLPs affects chlorophyll content in rice. **(A and B)** The Chla and Chlb content in the roots and shoots of two-week-old seedlings treated with 3 or 10 μmol L $^{-1}$ $CuSO_4$. **(C and D)** The Chla and Chlb content in the roots and shoots of two-week-old seedlings treated with 5 or 25 μmol L^{-1} $CdCl_2$ for 5 days. Values are the mean ± SD; n = 3. Different letters indicate a difference of $p \leq 0.05$ by the LSD test.

TABLE S1 | Primers used for plasmid construction.

Primer name	Primer sequence (5'-3')
gRNA1-F	GGCATAGCCCTCTCCAAGACTTTT
gRNA1-R	AAACAAAAGTCTTGGAGAGGGCTA
gRNA2-F	GGCATCCTGCTCCTCGCCACTCTT
gRNA2-R	AAACAAGAGTGGCGAGGAGCAGGA
CriOsGLP8-2F	CATCTCAAACACACCACCTGA
CriOsGLP8-2R	AAGACATTGACCAAAGTGA
CriOsGLP8-11F	CATCTCAAACACACCACCTGA
CriOsGLP8-11R	TTGCTGCCTTGAAGAAGTGAT
OsGLP8-2OE-F	GTGTTACTTCTGCAGGGTACCATGGCTTCACCATCTTCCTTCT
OsGLP8-2OE-R	ATAACGCGTACTAGTAAGCTTTTAGTAGTGGTTGTTCTCCCAA

TABLE S2 | Primers used for quantitative real-time PCR analysis.

Primer name	Primer sequence (5’-3’)
ACTIN1-F	TATGCTCTCCCCCATGCTATC
ACTIN1-R	CCGTTGTGGTGAATGAGTAACC
OsGLP8-2F	AGGCAGCCATGCTCGATAAG
OsGLP8-2R	GGGTGCAAAGTCAAGACGTG
OsGLP8-3F	TGTGCATCCTCGTGCTACTG
OsGLP8-3R	ACCCTTATGCACCACCTTGG
OsGLP8-5F	ACCTGTGCGTGTCAATGGAT
OsGLP8-5R	ACGTTGGACCCAACCTTGTT
OsGLP8-7F	GGCTTCACCATCTTCCTTCTGC
OsGLP8-7R	TGAGTGCTTATCGGCAACGC
OsGLP8-11F	ACTCACCTGCAGTGCTTGTC
OsGLP8-11R	TGTTCGTCTTCCGAGGAGTG
PAL-F	ACACAACTCCTCCACCTCCATCG
PAL-R	GCTCCTCCGCCGCCTTCC
C4H-F	GCACGCCATACTACACCAGAC
C4H-R	GTCGTCGTTGCCGCTCATC
4CL-F	CGACCGCCACGACCTCTC
4CL-R	GCCCTGCCTCCGTCATCC
CCoAoMT-F	CACCAACAAGACGCTGCTCAAG
CCoAoMT-R	CTCATCCGCCGACGACTGC
